# Bis(4-amino­pyridine){2,2′-[1,2-phenyl­enebis(nitrilo­methanylyl­idene)]diphen­ol­ato}cobalt(III) nitrate

**DOI:** 10.1107/S1600536813020953

**Published:** 2013-08-17

**Authors:** Sivanesan Dharmalingam, Sungho Yoon

**Affiliations:** a Department of Bio & Nano Chemistry, College of Natural Sciences, Kookmin University, 861-1 Jeongneung-dong, Seongbuk-gu, Seoul 136-702, Republic of Korea

## Abstract

In the title compound, [Co(C_20_H_14_N_2_O_2_)(C_5_H_6_N_2_)_2_]NO_3_, the Co^III^ atom is coordinated in a slightly elongated octa­hedral geometry by the N_2_O_2_ donor set of the tetra­dentate Schiff base ligand and by the pyridine N atoms of two *trans*-arranged monodentate 4-amino­pyridine mol­ecules. The pyridine rings are aligned nearly perpendicularly to each other [dihedral angle = 82.28 (13)°]. The phen­oxy rings form dihedral angles of 12.37 (12) and 12.16 (14)° with the phenyl­ene ring. In the crystal, N—H⋯O and C—H⋯O hydrogen bonds link the ions into a three-dimensional network.

## Related literature

For transition metal Schiff-base complexes with a tetra­dentate N_2_O_2_ ligand, see: Schenk *et al.* (2007 ▶); Yamada *et al.* (1999 ▶); Polson *et al.* (1997 ▶); Hirota *et al.* (1998 ▶). For related cobalt complexes, see: Amirnasr *et al.* (2001 ▶); Khandar *et al.* (2007 ▶); Salehi *et al.* (2009 ▶). For related dimeric cobalt complexes, see: Shimakoshi *et al.* (2005 ▶).
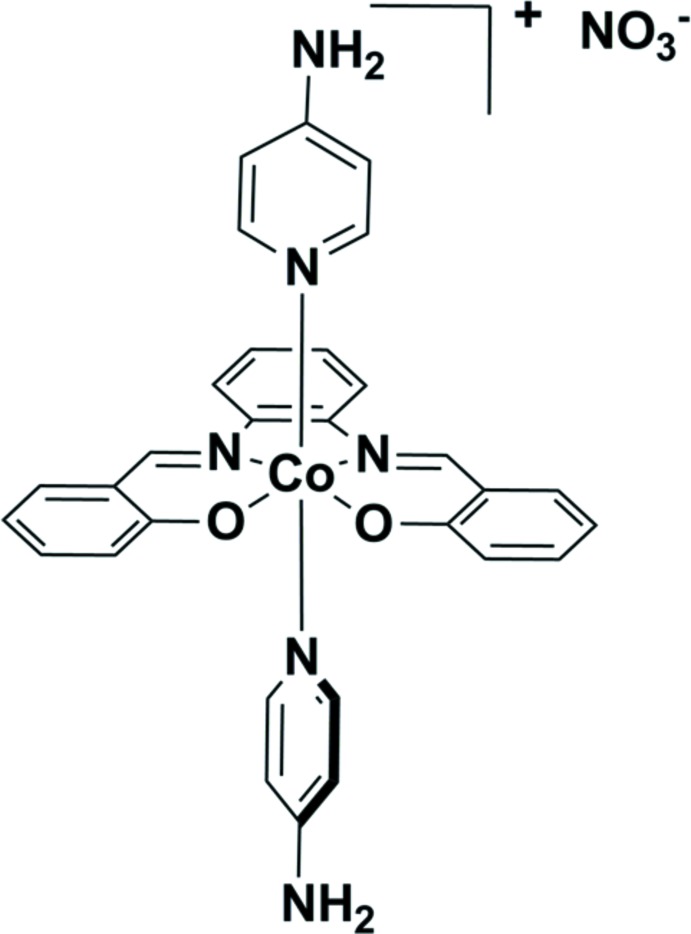


## Experimental

### 

#### Crystal data

[Co(C_20_H_14_N_2_O_2_)(C_5_H_6_N_2_)_2_]NO_3_
*M* *_r_* = 623.51
Monoclinic, 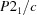

*a* = 13.072 (3) Å
*b* = 15.136 (3) Å
*c* = 17.192 (6) Å
β = 125.14 (2)°
*V* = 2781.6 (15) Å^3^
*Z* = 4
Mo *K*α radiation
μ = 0.67 mm^−1^
*T* = 293 K
0.21 × 0.17 × 0.11 mm

#### Data collection

Bruker SMART CCD area-detector diffractometer
Absorption correction: multi-scan (*SADABS*; Bruker, 2000 ▶) *T*_min_ = 0.826, *T*_max_ = 1.00
20500 measured reflections
6917 independent reflections
2486 reflections with *I* > 2σ(*I*)
*R*_int_ = 0.110

#### Refinement

*R*[*F* ^2^ > 2σ(*F* ^2^)] = 0.050
*wR*(*F* ^2^) = 0.084
*S* = 0.71
6917 reflections
404 parameters
2 restraints
H atoms treated by a mixture of independent and constrained refinement
Δρ_max_ = 0.79 e Å^−3^
Δρ_min_ = −0.70 e Å^−3^

### 

Data collection: *SMART* (Bruker, 2000 ▶); cell refinement: *SAINT* (Bruker, 2000 ▶); data reduction: *SAINT*; program(s) used to solve structure: *SHELXS97* (Sheldrick, 2008 ▶); program(s) used to refine structure: *SHELXL97* (Sheldrick, 2008 ▶); molecular graphics: *SHELXTL* (Sheldrick, 2008 ▶); software used to prepare material for publication: *SHELXTL*.

## Supplementary Material

Crystal structure: contains datablock(s) I, global. DOI: 10.1107/S1600536813020953/rz5078sup1.cif

Structure factors: contains datablock(s) I. DOI: 10.1107/S1600536813020953/rz5078Isup2.hkl

Additional supplementary materials:  crystallographic information; 3D view; checkCIF report

## Figures and Tables

**Table 1:** Hydrogen-bond geometry (Å, °)

*D*—H⋯*A*	*D*—H	H⋯*A*	*D*⋯*A*	*D*—H⋯*A*
C9—H9⋯O3	0.93	2.55	3.470 (4)	169
C26—H26⋯O3^i^	0.93	2.52	3.171 (5)	128
C44—H44⋯O3	0.93	2.36	3.217 (5)	152
C46—H46⋯O5	0.93	2.56	3.388 (5)	148
N5—H51*A*⋯O4^ii^	0.86 (2)	2.44 (3)	3.269 (5)	162 (4)
N5—H51*A*⋯O5^ii^	0.86 (2)	2.36 (3)	3.078 (5)	142 (4)
N5—H52*A*⋯O4^iii^	0.87 (2)	2.02 (2)	2.868 (5)	166 (3)

Symmetry codes: (i) 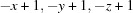; (ii) 
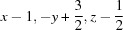
; (iii) 
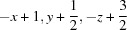
.

**Fig. 1.**
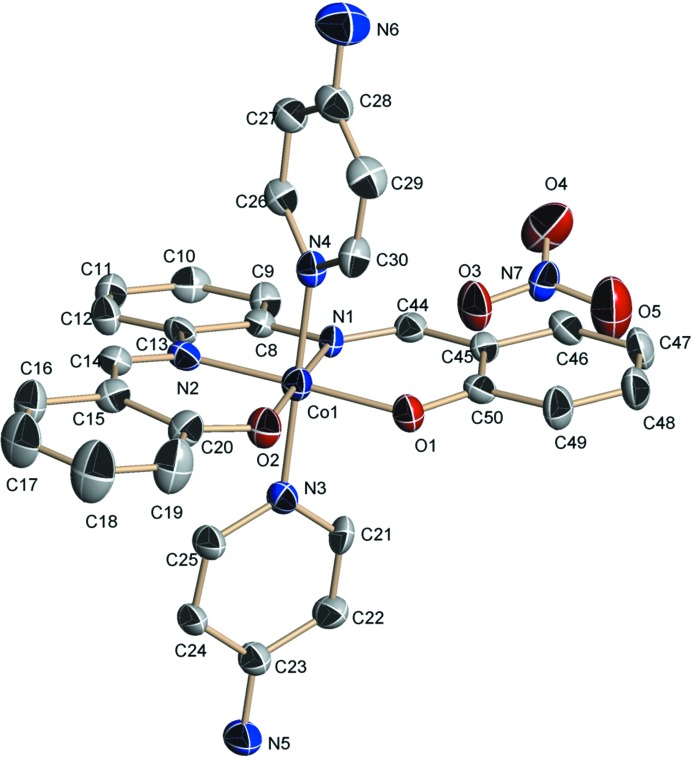
The molecular structure of the title compound showing displacement ellipsoids drawn at the 50% probability level. H atoms are omitted for clarity.

**Fig. 2.**
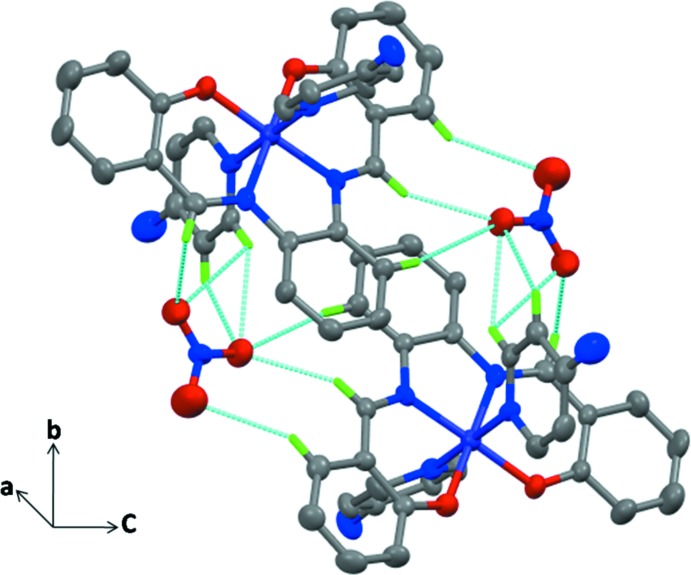
Partial packing diagram of the title compound showing the hydrogen bonding network (dotted lines).

## supplementary crystallographic information

### 1. Comment

Cobalt complexes containing Schiff base ligands are interesting synthetic models for cobalamine (B_12_) enzymes (Polson *et al.*, 1997; Hirota *et al.*, 1998). Among the various Schiff base ligated metal complexes, cobalt complexes with two axial amines show antimicrobial activities (Amirnasr *et al.*, 2001; Khandar *et al.*, 2007; Salehi *et al.*, 2009). Herein we report the synthesis and crystal structure of a new octahedrally coordinated cobalt(III) complex.

The molecular structure of the title compound is shown in Fig. 1. The equatorial positions of the slightly elongated octahedron are occupied by two oxygen and two nitrogen atoms of the Schiff base, and the axial positions by the nitrogen atoms of two 4-aminopyridine molecules. A nitrate anion is present in the lattice to balance the charge of the complex. The pyridine rings are mutually nearly perpendicular to each other, forming a dihedral angle of 82.28 (13)°. The distances between cobalt and the axial nitrogen atoms (1.960 (3) and 1.959 (3)Å) are longer than the equatorial Co—N bonds (1.890 (3) Å), and are comparable with the corresponding values observed in {[Co(III)(salophen)(dipyridine)]ClO_4_}, where salophen is 2,2'-[*o*-phenylenebis(nitrilomethylidyne)]diphenolato (Salehi *et al.*, 2009). In the Schiff base ligand, the C15–C20 and C45–C50 phenoxy rings are tilted with respect to the phenylene ring by 12.37 (12) and 12.16 (14)° respectively. In the crystal structure (Fig. 2), the ions are linked into a three-dimensional network by interionic N—H···O and C—H···O hydrogen bonding interactions (Table 1).

### 2. Experimental

To a mixture of the Schiff base ligand (0.108 g, 0.343 mmol ) and triethylamine (0.105 mL) in MeOH, (Co(NO_3_)_2_.6H_2_O (0.100 g, 0.343 mmol) was added and heated to 70°C. After two hours, 4-aminopyridine (0.064 g, 0.680 mmol) was added and heating and stirring continued for two hours. The resulting dark reddish-brown solid was crystallized in acetonitrile-diethyl ether (1:1 *v*/*v*) by diffusion method. Yield = 0.090 g (47 %). ^1^H-NMR (DMSO-d_6_, δ): 8.87 (s, 2H), 8.30 (d, *J* = 5.7, 4H), 7.41 (m, 2H), 7.30 (m, 4H), 7.15 (m, 2H), 6.81 (m, 4H), 6.26 (d, *J* = 5.7, 4H). FT-IR (KBr, cm^-1^): 3470 (m), 3393(w), 3364(m), 3203(s), 3062(w), 3020(w), 2966(w), 1630 (s), 1607(s), 1571(s), 1517(s), 1488(m), 1464(m), 1436(m), 1329(s), 1204(s), 1181(m), 1145(s), 1056(m), 1021(m), 968(w), 920(w), 826(m), 749(s), 566(w), 542(m).

### 3. Refinement

Hydrogen atoms bound to N were located in a difference Fourier map and refined isotropically with distance restraints (N—H = 0.86-0.87 Å) and *U*_iso_(H) = 1.5*U*_eq_(N). All other H atoms were introduced at their calculated positions and were then refined as riding, with C—H = 0.93 Å and *U*_iso_(H) = 1.2*U*_eq_(N) for aromatic H atoms. The crystals of the title compound systematically diffracted very poorly, resulting in a low observed/unique reflection ratio (36%).

### Crystal data

**Table:**

[Co(C_20_H_14_N_2_O_2_)(C_5_H_6_N_2_)_2_]NO_3_	*F*(000) = 1288
*M**_r_* = 623.51	*D*_x_ = 1.489 Mg m^−^^3^
Monoclinic, *P*2_1_/*c*	Mo *K*α radiation, λ = 0.71073 Å
Hall symbol: -P 2ybc	Cell parameters from 2630 reflections
*a* = 13.072 (3) Å	θ = 2.3–24.3°
*b* = 15.136 (3) Å	µ = 0.67 mm^−^^1^
*c* = 17.192 (6) Å	*T* = 293 K
β = 125.14 (2)°	Block, red
*V* = 2781.6 (15) Å^3^	0.21 × 0.17 × 0.11 mm
*Z* = 4	

### Data collection

**Table:**

Bruker SMART CCD area-detector diffractometer	6917 independent reflections
Radiation source: fine-focus sealed tube	2486 reflections with *I* > 2σ(*I*)
Graphite monochromator	*R*_int_ = 0.110
φ and ω scans	θ_max_ = 28.3°, θ_min_ = 1.9°
Absorption correction: multi-scan (*SADABS*; Bruker, 2000)	*h* = −17→17
*T*_min_ = 0.826, *T*_max_ = 1.00	*k* = −20→17
20500 measured reflections	*l* = −22→19

### Refinement

**Table:**

Refinement on *F*^2^	Primary atom site location: structure-invariant direct methods
Least-squares matrix: full	Secondary atom site location: difference Fourier map
*R*[*F*^2^ > 2σ(*F*^2^)] = 0.050	Hydrogen site location: inferred from neighbouring sites
*wR*(*F*^2^) = 0.084	H atoms treated by a mixture of independent and constrained refinement
*S* = 0.71	*w* = 1/[σ^2^(*F*_o_^2^) + (0.*P*)^2^] where *P* = (*F*_o_^2^ + 2*F*_c_^2^)/3
6917 reflections	(Δ/σ)_max_ = 0.001
404 parameters	Δρ_max_ = 0.79 e Å^−^^3^
2 restraints	Δρ_min_ = −0.70 e Å^−^^3^

### Special details

**Table:**

Geometry. All esds (except the esd in the dihedral angle between two l.s. planes) are estimated using the full covariance matrix. The cell esds are taken into account individually in the estimation of esds in distances, angles and torsion angles; correlations between esds in cell parameters are only used when they are defined by crystal symmetry. An approximate (isotropic) treatment of cell esds is used for estimating esds involving l.s. planes.
Refinement. Refinement of F^2^ against ALL reflections. The weighted R-factor wR and goodness of fit S are based on F^2^, conventional R-factors R are based on F, with F set to zero for negative F^2^. The threshold expression of F^2^ > 2sigma(F^2^) is used only for calculating R-factors(gt) etc. and is not relevant to the choice of reflections for refinement. R-factors based on F^2^ are statistically about twice as large as those based on F, and R- factors based on ALL data will be even larger.

### Fractional atomic coordinates and isotropic or equivalent isotropic displacement parameters (Å^2^)

**Table:**

	*x*	*y*	*z*	*U*_iso_*/*U*_eq_	
Co1	0.36975 (4)	0.75435 (4)	0.33861 (3)	0.02774 (14)	
O1	0.5005 (2)	0.83721 (16)	0.40079 (16)	0.0318 (7)	
O2	0.3029 (2)	0.83328 (16)	0.23490 (16)	0.0338 (7)	
N1	0.4376 (2)	0.67753 (19)	0.44404 (19)	0.0241 (8)	
N2	0.2362 (3)	0.6726 (2)	0.2758 (2)	0.0260 (8)	
N3	0.2806 (3)	0.8170 (2)	0.3824 (2)	0.0282 (8)	
N4	0.4594 (3)	0.6910 (2)	0.29547 (19)	0.0273 (8)	
N5	0.0804 (4)	0.9424 (3)	0.4717 (3)	0.0423 (11)	
N6	0.6508 (5)	0.5547 (4)	0.2043 (3)	0.0591 (14)	
C8	0.3526 (3)	0.6096 (2)	0.4287 (2)	0.0244 (9)	
C9	0.3708 (3)	0.5500 (2)	0.4972 (3)	0.0305 (10)	
H9	0.4429	0.5526	0.5589	0.037*	
C10	0.2805 (3)	0.4866 (3)	0.4726 (3)	0.0329 (10)	
H10	0.2934	0.4456	0.5177	0.039*	
C11	0.1713 (3)	0.4837 (3)	0.3816 (3)	0.0346 (11)	
H11	0.1106	0.4414	0.3659	0.041*	
C12	0.1533 (3)	0.5433 (2)	0.3149 (2)	0.0307 (10)	
H12	0.0800	0.5412	0.2538	0.037*	
C13	0.2432 (3)	0.6070 (3)	0.3372 (2)	0.0256 (9)	
C14	0.1437 (3)	0.6764 (3)	0.1850 (2)	0.0297 (10)	
H14	0.0880	0.6291	0.1597	0.036*	
C15	0.1218 (3)	0.7465 (3)	0.1223 (2)	0.0291 (9)	
C16	0.0092 (3)	0.7426 (3)	0.0285 (2)	0.0420 (11)	
H16	−0.0428	0.6936	0.0103	0.050*	
C17	−0.0232 (4)	0.8088 (3)	−0.0344 (3)	0.0529 (14)	
H17	−0.0970	0.8054	−0.0952	0.063*	
C18	0.0543 (4)	0.8822 (3)	−0.0077 (3)	0.0660 (16)	
H18	0.0323	0.9274	−0.0513	0.079*	
C19	0.1629 (4)	0.8887 (3)	0.0820 (3)	0.0520 (13)	
H19	0.2130	0.9384	0.0983	0.062*	
C20	0.1996 (3)	0.8220 (3)	0.1496 (3)	0.0324 (10)	
C21	0.3351 (3)	0.8436 (2)	0.4730 (2)	0.0351 (11)	
H21	0.4208	0.8347	0.5160	0.042*	
C22	0.2729 (3)	0.8826 (3)	0.5055 (3)	0.0344 (11)	
H22	0.3162	0.8986	0.5692	0.041*	
C23	0.1447 (3)	0.8989 (3)	0.4443 (3)	0.0294 (10)	
C24	0.0876 (3)	0.8706 (2)	0.3502 (2)	0.0306 (10)	
H24	0.0020	0.8781	0.3059	0.037*	
C25	0.1568 (3)	0.8324 (3)	0.3237 (2)	0.0334 (10)	
H25	0.1158	0.8155	0.2604	0.040*	
C26	0.4613 (3)	0.6024 (3)	0.2911 (2)	0.0288 (10)	
H26	0.4171	0.5707	0.3091	0.035*	
C27	0.5235 (3)	0.5549 (3)	0.2622 (2)	0.0325 (10)	
H27	0.5208	0.4935	0.2610	0.039*	
C28	0.5905 (4)	0.6002 (3)	0.2347 (3)	0.0367 (11)	
C29	0.5908 (4)	0.6917 (3)	0.2400 (3)	0.0384 (11)	
H29	0.6358	0.7246	0.2236	0.046*	
C30	0.5258 (3)	0.7341 (3)	0.2689 (2)	0.0347 (10)	
H30	0.5271	0.7955	0.2705	0.042*	
C44	0.5503 (3)	0.6828 (2)	0.5221 (2)	0.0266 (9)	
H44	0.5756	0.6389	0.5676	0.032*	
C45	0.6375 (3)	0.7514 (3)	0.5425 (2)	0.0248 (8)	
C46	0.7566 (3)	0.7467 (3)	0.6315 (2)	0.0328 (9)	
H46	0.7752	0.6986	0.6713	0.039*	
C47	0.8436 (3)	0.8113 (3)	0.6593 (3)	0.0383 (11)	
H47	0.9207	0.8079	0.7179	0.046*	
C48	0.8154 (3)	0.8826 (3)	0.5988 (3)	0.0438 (12)	
H48	0.8748	0.9267	0.6172	0.053*	
C49	0.7023 (3)	0.8888 (3)	0.5131 (3)	0.0379 (11)	
H49	0.6864	0.9369	0.4740	0.045*	
C50	0.6088 (3)	0.8243 (3)	0.4824 (3)	0.0269 (10)	
O3	0.6426 (2)	0.59009 (18)	0.72076 (18)	0.0473 (8)	
O5	0.8220 (3)	0.6406 (2)	0.8288 (2)	0.0772 (11)	
O4	0.7833 (3)	0.5069 (2)	0.83658 (19)	0.0651 (10)	
N25	0.7482 (3)	0.5781 (2)	0.7972 (2)	0.0341 (9)	
H50A	0.694 (3)	0.581 (3)	0.190 (3)	0.052 (16)*	
H49A	0.646 (4)	0.4993 (19)	0.201 (3)	0.08 (2)*	
H52A	0.112 (3)	0.957 (2)	0.5305 (17)	0.041 (14)*	
H51A	0.000 (2)	0.943 (3)	0.440 (2)	0.070 (17)*	

### Atomic displacement parameters (Å^2^)

**Table:**

	*U*^11^	*U*^22^	*U*^33^	*U*^12^	*U*^13^	*U*^23^
Co1	0.0255 (3)	0.0291 (3)	0.0242 (3)	−0.0007 (3)	0.0117 (2)	0.0000 (3)
O1	0.0255 (15)	0.0332 (18)	0.0296 (16)	−0.0010 (13)	0.0117 (13)	0.0021 (14)
O2	0.0329 (15)	0.0356 (18)	0.0245 (15)	−0.0007 (14)	0.0118 (13)	0.0053 (14)
N1	0.0184 (17)	0.028 (2)	0.0200 (17)	−0.0034 (15)	0.0074 (14)	−0.0052 (15)
N2	0.0246 (18)	0.030 (2)	0.0220 (18)	0.0071 (16)	0.0129 (15)	0.0025 (16)
N3	0.0247 (18)	0.025 (2)	0.0253 (18)	0.0041 (16)	0.0089 (15)	0.0037 (16)
N4	0.0257 (18)	0.025 (2)	0.0263 (19)	−0.0034 (16)	0.0120 (15)	0.0042 (16)
N5	0.034 (3)	0.055 (3)	0.038 (3)	0.005 (2)	0.021 (2)	−0.011 (2)
N6	0.067 (3)	0.058 (4)	0.081 (4)	0.004 (3)	0.059 (3)	0.003 (3)
C8	0.023 (2)	0.024 (2)	0.026 (2)	0.0028 (19)	0.0146 (18)	0.0009 (19)
C9	0.026 (2)	0.036 (3)	0.028 (2)	−0.004 (2)	0.0145 (19)	0.001 (2)
C10	0.041 (3)	0.029 (3)	0.038 (3)	0.000 (2)	0.028 (2)	0.006 (2)
C11	0.029 (2)	0.033 (3)	0.035 (3)	−0.005 (2)	0.015 (2)	−0.004 (2)
C12	0.024 (2)	0.035 (3)	0.024 (2)	−0.001 (2)	0.0085 (18)	0.005 (2)
C13	0.022 (2)	0.026 (2)	0.024 (2)	0.0083 (19)	0.0107 (18)	0.0052 (19)
C14	0.026 (2)	0.029 (3)	0.032 (2)	0.004 (2)	0.015 (2)	−0.002 (2)
C15	0.034 (2)	0.029 (2)	0.024 (2)	0.002 (2)	0.0167 (18)	0.002 (2)
C16	0.035 (2)	0.047 (3)	0.028 (2)	−0.007 (3)	0.0094 (19)	−0.003 (3)
C17	0.049 (3)	0.058 (4)	0.024 (3)	−0.001 (3)	0.005 (2)	0.015 (3)
C18	0.067 (4)	0.068 (4)	0.037 (3)	−0.011 (3)	0.014 (3)	0.014 (3)
C19	0.055 (3)	0.046 (3)	0.038 (3)	−0.013 (3)	0.017 (2)	0.009 (3)
C20	0.037 (3)	0.030 (3)	0.024 (2)	0.000 (2)	0.014 (2)	0.003 (2)
C21	0.024 (2)	0.038 (3)	0.024 (2)	−0.004 (2)	0.0025 (19)	−0.010 (2)
C22	0.035 (3)	0.038 (3)	0.026 (2)	0.004 (2)	0.015 (2)	−0.005 (2)
C23	0.027 (2)	0.031 (3)	0.028 (2)	0.002 (2)	0.014 (2)	0.001 (2)
C24	0.021 (2)	0.036 (3)	0.024 (2)	0.006 (2)	0.0067 (18)	−0.002 (2)
C25	0.027 (2)	0.042 (3)	0.023 (2)	0.006 (2)	0.0094 (19)	−0.002 (2)
C26	0.028 (2)	0.035 (3)	0.022 (2)	0.003 (2)	0.0133 (19)	0.006 (2)
C27	0.031 (2)	0.036 (3)	0.030 (2)	0.002 (2)	0.017 (2)	0.000 (2)
C28	0.032 (3)	0.047 (3)	0.030 (3)	0.007 (2)	0.017 (2)	0.005 (2)
C29	0.040 (3)	0.049 (3)	0.037 (3)	−0.001 (2)	0.028 (2)	0.006 (2)
C30	0.033 (2)	0.038 (3)	0.030 (2)	−0.009 (2)	0.0167 (19)	−0.003 (2)
C44	0.028 (2)	0.028 (3)	0.024 (2)	0.002 (2)	0.0153 (19)	−0.0018 (19)
C45	0.0214 (19)	0.025 (2)	0.029 (2)	−0.002 (2)	0.0147 (17)	−0.002 (2)
C46	0.025 (2)	0.039 (3)	0.033 (2)	0.005 (2)	0.0166 (18)	0.003 (2)
C47	0.018 (2)	0.044 (3)	0.037 (3)	−0.003 (2)	0.0072 (19)	0.000 (2)
C48	0.023 (2)	0.038 (3)	0.051 (3)	−0.007 (2)	0.010 (2)	−0.004 (2)
C49	0.028 (2)	0.036 (3)	0.041 (3)	−0.010 (2)	0.015 (2)	0.000 (2)
C50	0.025 (2)	0.027 (3)	0.030 (2)	0.004 (2)	0.0161 (19)	0.001 (2)
O3	0.0328 (17)	0.050 (2)	0.0330 (17)	−0.0014 (16)	0.0039 (14)	0.0093 (16)
O5	0.054 (2)	0.074 (3)	0.065 (2)	−0.014 (2)	0.0121 (18)	0.006 (2)
O4	0.085 (2)	0.038 (2)	0.040 (2)	0.0086 (19)	0.0172 (18)	0.0079 (17)
N25	0.034 (2)	0.030 (2)	0.033 (2)	−0.0099 (19)	0.0157 (19)	−0.0033 (19)

### Geometric parameters (Å, º)

**Table:**

Co1—O1	1.880 (2)	C17—C18	1.391 (5)
Co1—N2	1.890 (3)	C17—H17	0.9300
Co1—N1	1.890 (3)	C18—C19	1.372 (5)
Co1—O2	1.891 (2)	C18—H18	0.9300
Co1—N3	1.959 (3)	C19—C20	1.399 (5)
Co1—N4	1.960 (3)	C19—H19	0.9300
O1—C50	1.312 (4)	C21—C22	1.358 (4)
O2—C20	1.313 (4)	C21—H21	0.9300
N1—C44	1.304 (4)	C22—C23	1.395 (4)
N1—C8	1.423 (4)	C22—H22	0.9300
N2—C14	1.316 (4)	C23—C24	1.402 (4)
N2—C13	1.414 (4)	C24—C25	1.356 (4)
N3—C25	1.346 (4)	C24—H24	0.9300
N3—C21	1.348 (4)	C25—H25	0.9300
N4—C26	1.344 (4)	C26—C27	1.375 (5)
N4—C30	1.358 (4)	C26—H26	0.9300
N5—C23	1.348 (5)	C27—C28	1.392 (5)
N5—H52A	0.87 (2)	C27—H27	0.9300
N5—H51A	0.86 (2)	C28—C29	1.387 (5)
N6—C28	1.357 (5)	C29—C30	1.367 (5)
N6—H50A	0.83 (3)	C29—H29	0.9300
N6—H49A	0.84 (3)	C30—H30	0.9300
C8—C13	1.390 (4)	C44—C45	1.429 (5)
C8—C9	1.391 (5)	C44—H44	0.9300
C9—C10	1.384 (5)	C45—C50	1.407 (5)
C9—H9	0.9300	C45—C46	1.424 (4)
C10—C11	1.385 (4)	C46—C47	1.361 (5)
C10—H10	0.9300	C46—H46	0.9300
C11—C12	1.369 (5)	C47—C48	1.394 (5)
C11—H11	0.9300	C47—H47	0.9300
C12—C13	1.392 (5)	C48—C49	1.363 (4)
C12—H12	0.9300	C48—H48	0.9300
C14—C15	1.420 (5)	C49—C50	1.408 (5)
C14—H14	0.9300	C49—H49	0.9300
C15—C20	1.417 (5)	O3—N25	1.256 (4)
C15—C16	1.428 (4)	O5—N25	1.231 (4)
C16—C17	1.350 (5)	O4—N25	1.214 (4)
C16—H16	0.9300		
			
O1—Co1—N2	178.95 (12)	C18—C17—H17	120.2
O1—Co1—N1	95.39 (11)	C19—C18—C17	120.9 (4)
N2—Co1—N1	85.08 (13)	C19—C18—H18	119.5
O1—Co1—O2	83.70 (11)	C17—C18—H18	119.5
N2—Co1—O2	95.82 (12)	C18—C19—C20	121.4 (4)
N1—Co1—O2	178.78 (12)	C18—C19—H19	119.3
O1—Co1—N3	90.33 (12)	C20—C19—H19	119.3
N2—Co1—N3	88.74 (12)	O2—C20—C19	118.3 (4)
N1—Co1—N3	89.07 (12)	O2—C20—C15	123.7 (4)
O2—Co1—N3	90.13 (12)	C19—C20—C15	117.9 (3)
O1—Co1—N4	89.75 (12)	N3—C21—C22	124.1 (3)
N2—Co1—N4	91.18 (12)	N3—C21—H21	117.9
N1—Co1—N4	90.52 (12)	C22—C21—H21	117.9
O2—Co1—N4	90.28 (12)	C21—C22—C23	120.8 (4)
N3—Co1—N4	179.59 (13)	C21—C22—H22	119.6
C50—O1—Co1	125.2 (2)	C23—C22—H22	119.6
C20—O2—Co1	126.2 (3)	N5—C23—C22	122.9 (4)
C44—N1—C8	122.3 (3)	N5—C23—C24	121.8 (4)
C44—N1—Co1	125.3 (3)	C22—C23—C24	115.2 (4)
C8—N1—Co1	112.4 (2)	C25—C24—C23	120.3 (3)
C14—N2—C13	122.5 (3)	C25—C24—H24	119.9
C14—N2—Co1	124.4 (3)	C23—C24—H24	119.9
C13—N2—Co1	113.1 (2)	N3—C25—C24	124.6 (4)
C25—N3—C21	115.1 (3)	N3—C25—H25	117.7
C25—N3—Co1	121.3 (3)	C24—C25—H25	117.7
C21—N3—Co1	123.5 (2)	N4—C26—C27	125.2 (4)
C26—N4—C30	115.1 (3)	N4—C26—H26	117.4
C26—N4—Co1	122.9 (3)	C27—C26—H26	117.4
C30—N4—Co1	122.0 (3)	C26—C27—C28	118.9 (4)
C23—N5—H52A	123 (2)	C26—C27—H27	120.5
C23—N5—H51A	124 (3)	C28—C27—H27	120.5
H52A—N5—H51A	110 (3)	N6—C28—C29	123.4 (4)
C28—N6—H50A	121 (3)	N6—C28—C27	119.9 (5)
C28—N6—H49A	120 (3)	C29—C28—C27	116.6 (4)
H50A—N6—H49A	119 (4)	C30—C29—C28	120.9 (4)
C13—C8—C9	120.2 (4)	C30—C29—H29	119.6
C13—C8—N1	114.5 (3)	C28—C29—H29	119.6
C9—C8—N1	125.3 (3)	N4—C30—C29	123.3 (4)
C10—C9—C8	119.4 (3)	N4—C30—H30	118.4
C10—C9—H9	120.3	C29—C30—H30	118.4
C8—C9—H9	120.3	N1—C44—C45	124.5 (4)
C9—C10—C11	120.7 (4)	N1—C44—H44	117.7
C9—C10—H10	119.6	C45—C44—H44	117.7
C11—C10—H10	119.6	C50—C45—C46	119.6 (4)
C12—C11—C10	119.5 (4)	C50—C45—C44	123.5 (3)
C12—C11—H11	120.2	C46—C45—C44	116.8 (4)
C10—C11—H11	120.2	C47—C46—C45	121.1 (4)
C11—C12—C13	121.0 (3)	C47—C46—H46	119.4
C11—C12—H12	119.5	C45—C46—H46	119.4
C13—C12—H12	119.5	C46—C47—C48	119.1 (3)
C8—C13—C12	119.1 (4)	C46—C47—H47	120.5
C8—C13—N2	114.0 (3)	C48—C47—H47	120.5
C12—C13—N2	126.8 (3)	C49—C48—C47	121.1 (4)
N2—C14—C15	125.4 (4)	C49—C48—H48	119.5
N2—C14—H14	117.3	C47—C48—H48	119.5
C15—C14—H14	117.3	C48—C49—C50	121.7 (4)
C20—C15—C14	124.0 (3)	C48—C49—H49	119.2
C20—C15—C16	118.9 (4)	C50—C49—H49	119.2
C14—C15—C16	116.9 (4)	O1—C50—C45	124.7 (3)
C17—C16—C15	121.2 (4)	O1—C50—C49	117.8 (4)
C17—C16—H16	119.4	C45—C50—C49	117.4 (3)
C15—C16—H16	119.4	O4—N25—O5	119.2 (4)
C16—C17—C18	119.6 (4)	O4—N25—O3	123.1 (4)
C16—C17—H17	120.2	O5—N25—O3	117.4 (4)
			
N1—Co1—O1—C50	13.0 (3)	C14—N2—C13—C12	−5.4 (6)
O2—Co1—O1—C50	−167.8 (3)	Co1—N2—C13—C12	173.1 (3)
N3—Co1—O1—C50	102.1 (3)	C13—N2—C14—C15	171.2 (3)
N4—Co1—O1—C50	−77.5 (3)	Co1—N2—C14—C15	−7.2 (5)
O1—Co1—O2—C20	177.3 (3)	N2—C14—C15—C20	1.4 (6)
N2—Co1—O2—C20	−3.7 (3)	N2—C14—C15—C16	−173.8 (3)
N3—Co1—O2—C20	−92.4 (3)	C20—C15—C16—C17	0.7 (6)
N4—Co1—O2—C20	87.5 (3)	C14—C15—C16—C17	176.2 (4)
O1—Co1—N1—C44	−10.6 (3)	C15—C16—C17—C18	0.2 (7)
N2—Co1—N1—C44	170.3 (3)	C16—C17—C18—C19	−0.6 (8)
N3—Co1—N1—C44	−100.9 (3)	C17—C18—C19—C20	0.2 (8)
N4—Co1—N1—C44	79.2 (3)	Co1—O2—C20—C19	178.6 (3)
O1—Co1—N1—C8	171.1 (2)	Co1—O2—C20—C15	−0.4 (5)
N2—Co1—N1—C8	−8.0 (2)	C18—C19—C20—O2	−178.3 (4)
N3—Co1—N1—C8	80.8 (2)	C18—C19—C20—C15	0.7 (7)
N4—Co1—N1—C8	−99.1 (2)	C14—C15—C20—O2	2.8 (6)
N1—Co1—N2—C14	−173.5 (3)	C16—C15—C20—O2	177.8 (3)
O2—Co1—N2—C14	7.3 (3)	C14—C15—C20—C19	−176.2 (4)
N3—Co1—N2—C14	97.3 (3)	C16—C15—C20—C19	−1.1 (6)
N4—Co1—N2—C14	−83.1 (3)	C25—N3—C21—C22	0.5 (6)
N1—Co1—N2—C13	8.0 (2)	Co1—N3—C21—C22	−176.1 (3)
O2—Co1—N2—C13	−171.2 (2)	N3—C21—C22—C23	−0.9 (6)
N3—Co1—N2—C13	−81.2 (2)	C21—C22—C23—N5	−175.7 (4)
N4—Co1—N2—C13	98.4 (2)	C21—C22—C23—C24	1.3 (6)
O1—Co1—N3—C25	136.1 (3)	N5—C23—C24—C25	175.6 (4)
N2—Co1—N3—C25	−43.4 (3)	C22—C23—C24—C25	−1.5 (6)
N1—Co1—N3—C25	−128.5 (3)	C21—N3—C25—C24	−0.7 (6)
O2—Co1—N3—C25	52.4 (3)	Co1—N3—C25—C24	176.0 (3)
O1—Co1—N3—C21	−47.6 (3)	C23—C24—C25—N3	1.2 (6)
N2—Co1—N3—C21	132.9 (3)	C30—N4—C26—C27	−0.3 (5)
N1—Co1—N3—C21	47.8 (3)	Co1—N4—C26—C27	−179.4 (3)
O2—Co1—N3—C21	−131.3 (3)	N4—C26—C27—C28	−0.1 (6)
O1—Co1—N4—C26	141.8 (3)	C26—C27—C28—N6	−178.8 (4)
N2—Co1—N4—C26	−38.7 (3)	C26—C27—C28—C29	0.9 (5)
N1—Co1—N4—C26	46.4 (3)	N6—C28—C29—C30	178.3 (4)
O2—Co1—N4—C26	−134.5 (3)	C27—C28—C29—C30	−1.3 (6)
O1—Co1—N4—C30	−37.3 (3)	C26—N4—C30—C29	−0.2 (5)
N2—Co1—N4—C30	142.2 (3)	Co1—N4—C30—C29	178.9 (3)
N1—Co1—N4—C30	−132.7 (3)	C28—C29—C30—N4	1.1 (6)
O2—Co1—N4—C30	46.4 (3)	C8—N1—C44—C45	−177.6 (3)
C44—N1—C8—C13	−171.8 (3)	Co1—N1—C44—C45	4.3 (5)
Co1—N1—C8—C13	6.5 (4)	N1—C44—C45—C50	3.8 (6)
C44—N1—C8—C9	9.5 (6)	N1—C44—C45—C46	179.9 (3)
Co1—N1—C8—C9	−172.2 (3)	C50—C45—C46—C47	−0.4 (6)
C13—C8—C9—C10	1.7 (6)	C44—C45—C46—C47	−176.7 (3)
N1—C8—C9—C10	−179.7 (3)	C45—C46—C47—C48	−0.7 (6)
C8—C9—C10—C11	−1.7 (6)	C46—C47—C48—C49	0.6 (6)
C9—C10—C11—C12	0.9 (6)	C47—C48—C49—C50	0.6 (7)
C10—C11—C12—C13	−0.1 (6)	Co1—O1—C50—C45	−9.3 (5)
C9—C8—C13—C12	−0.9 (6)	Co1—O1—C50—C49	172.0 (3)
N1—C8—C13—C12	−179.6 (3)	C46—C45—C50—O1	−177.1 (3)
C9—C8—C13—N2	178.6 (3)	C44—C45—C50—O1	−1.1 (6)
N1—C8—C13—N2	−0.1 (5)	C46—C45—C50—C49	1.6 (5)
C11—C12—C13—C8	0.0 (6)	C44—C45—C50—C49	177.5 (3)
C11—C12—C13—N2	−179.4 (3)	C48—C49—C50—O1	177.1 (4)
C14—N2—C13—C8	175.1 (3)	C48—C49—C50—C45	−1.7 (6)
Co1—N2—C13—C8	−6.4 (4)		

### Hydrogen-bond geometry (Å, º)

**Table:**

*D*—H···*A*	*D*—H	H···*A*	*D*···*A*	*D*—H···*A*
C9—H9···O3	0.93	2.55	3.470 (4)	169
C26—H26···O3^i^	0.93	2.52	3.171 (5)	128
C44—H44···O3	0.93	2.36	3.217 (5)	152
C46—H46···O5	0.93	2.56	3.388 (5)	148
N5—H51*A*···O4^ii^	0.86 (2)	2.44 (3)	3.269 (5)	162 (4)
N5—H51*A*···O5^ii^	0.86 (2)	2.36 (3)	3.078 (5)	142 (4)
N5—H52*A*···O4^iii^	0.87 (2)	2.02 (2)	2.868 (5)	166 (3)

Symmetry codes: (i) −*x*+1, −*y*+1, −*z*+1; (ii) *x*−1, −*y*+3/2, *z*−1/2; (iii) −*x*+1, *y*+1/2, −*z*+3/2.
